# Novel xylanases from *Simplicillium obclavatum* MTCC 9604: comparative analysis of production, purification and characterization of enzyme from submerged and solid state fermentation

**DOI:** 10.1186/2193-1801-2-382

**Published:** 2013-08-14

**Authors:** Saugata Roy, Tanmay Dutta, Tuhin Subhra Sarkar, Sanjay Ghosh

**Affiliations:** Department of Biochemistry, University of Calcutta, 35, Ballygunge Circular Road, Kolkata, 700 019 West Bengal India; Department of Biochemistry and Molecular Biology, Miller School of Medicine, University of Miami, 1011 NW 15th Street, Miami, FL 33136 USA

**Keywords:** *Simplicillium obclavatum*, Xylanase, Solid state fermentation, Submerged fermentation

## Abstract

The production of extracellular xylanase by a newly isolated fungus *Simplicillium obclavatum* MTCC 9604 was studied in solid-state and submerged fermentation. Multiple xylanases and endoglucanases were produced by the strain during growth on wheat bran in solid state fermentation (SSF). A single xylanase isoform was found to be produced by the same fungus under submerged fermentation (SF) using wheat bran as sole carbon source. Enzyme activity, stability and the protein yield were much higher in SSF than SF. The two dimensional zymogram of the crude enzyme indicated the presence of six isoforms with different pI values starting from pH 3–10. The optimum temperature and pH for the partially purified xylanase activity were 50°C and pH 5.0 respectively; xylanase enzymes exhibited remarkable stability over a broad pH range and the temperature range of 30-60°C which has great potential to be used in biofuels, animal feed and food industry applications.

## Background

Xylanases (endo-1, 4-β-D-xylanohydrolase; EC 3.2.1.8) are glycosidases involved in depolymerization of xylan, the major renewable hemicellulosic polysaccharide of plant cell wall. The hemicelluloses represent an enormous reserve of utilizable biomass as hardwoods consist of 25-32% hemicelluloses whereas softwood contains only 15-25% hemicelluloses. Undoubtedly, hemicelluloses are the second most abundant renewable resource, only exceeded by cellulose. Xylanase is produced by bacteria (Gilbert and Hazlewood 1993; Kiddinamoorthy et al. 2008; Sanghi et al. 2008; Sunna and Antranikian 1997), fungi (Chavez et al. 2006; Chavez et al. 2002a, b; Liao et al. 2012; Liu et al. 2006; Nair et al. 2008; Okafor et al. 2007; Sunna and Antranikian 1997), actinomycetes (Ninawe et al. 2008) and yeast (Liu et al. 1998). Recently, interest in xylanase has markedly increased due to its wide variety of biotechnological applications such as pre-bleaching of pulp, improving the digestibility of the animal feed stocks, modification of cereal based stuffs, bioconversion of lignocellulosic material and agro waste to fermentable products, clarification of fruit juices and degumming of plant fibers (Kapoor et al. 2001; Kuhad and Singh 1993; Ratanakhanokchai et al. 1999) etc. The cellulase free xylanase active at high temperature and pH are gaining importance in pulp and paper industry as they reduce the need for toxic chlorinated compounds making the bleaching process environment friendly (Srinivasan and Rele 1999; Viikari et al. 1994).

Industrial purification of enzymes on large scale is associated mainly with substrate. The use of agriculture residues as low cost substrates for the production of industrial enzymes is a significant way to reduce production cost. The technique of fermentation using solid substrate has the great advantage over submerged fermentation (SF) due to absence or near absence of aqueous phase that provides natural habitat for growth of microorganisms. Other advantages are as follows: economy of the space, simplicity of the media, simple machinery, greater compactness of the fermentation vessel owing to a low water volume, greater product yields, reduced energy demand, lower capital and recurring expenditure in industries, easier scale up processes, lesser volume of solvent needed for product recovery, absence of foam build – up and easier control of contamination due to the low moisture level in the system (Archana and Satyanarayana 1997; Ayyachamy and Vatsala 2007).

The majority of xylanases utilized in biotechnology are still derived from well characterized bacteria and fungi. In this study, xylanase producing fungus *Simplicillium obclavatum* MTCC 9602 was isolated from soil. The main objective of this study is the comparative analysis of production, purification and characterization of xylanase enzyme from submerged and solid state fermentation. In order to investigate the biotechnological applications of this enzyme, it would be desirable to purify and characterize it.

This paper reports for the first time about the production and properties of multiple forms of xylanases from the *S. obclavatum* MTCC 9602. An attempt was made in optimizing a low cost solid state fermentation medium that could allow enhanced product recovery as well as high enzyme productivity. Production of xylanase was also compared with the submerged fermentations (SF). In the present study, optimum culture condition, isozyme pattern, pH optimum, temperature optimum, pH stability, thermal stability, two dimensional gel electrophoresis followed by zymogram analysis and the action of several chemicals on the partially purified enzyme from SSF culture and enzyme from SF culture have also been characterized.

## Results and discussion

### Optimization of culture conditions and production of xylanase in SSF and SF

**Figure 1:**
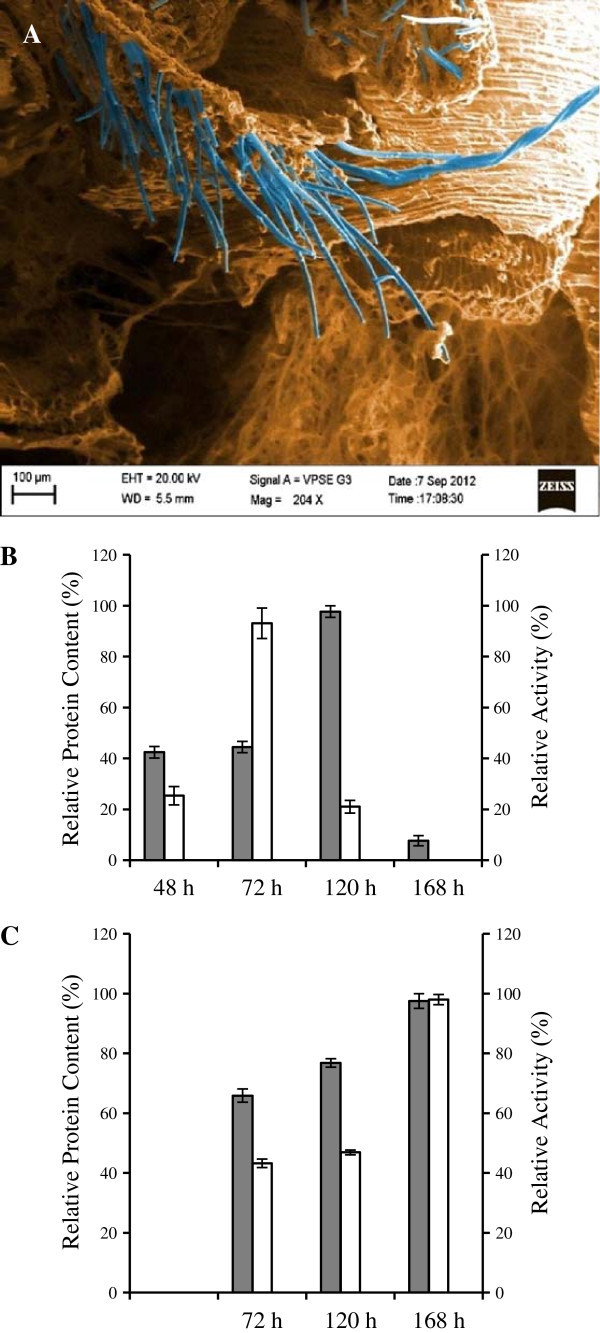
**Time dependent expression of xylanase of*S. obclavatum*in SSF and SF.** Panel **(A)** SEM Diagram of Fungus growing on wheat bran as sole source of carbon. Panel **(B)** Day wise production of relative protein content (% of maximum) (Dark Square) and relative xylanase activity (% of maximum) (Light Square) in submerged fermentation. Panel **(C)** Day wise production of relative protein content (% of maximum) (Dark Square) and relative xylanase activity (% of maximum) (Light Square) in solid state fermentation. The results are expressed in mean ± SD from n = 3.

The xylanase production has been reported from lot of fungal systems such as *Thermomyces lanuginosus*, *Thermoascus aurantiacus*, *Aspergillus awamori*, *A. niger*, *A. oryzae*, *Penicillium canescens*, *Penicillium citrinum*, *Ceriporiopsis subvermispora*, *Melanocarpus albomyces*, *P. thermophila* J18 and *Trichoderma reesei*. The substrates used mostly for xylanase production include wheat bran, corn cobs, sugarcane bagasse, bagasse pulp, spent sulphite liquor, rice straw, wheat straw, sorghum flour and eucalyptus pulp. However, lignocellulosic materials especially wheat bran has been more successful in production with higher titers being attributed to its hemicellulose nature, favorable degradability and the presence of some nutrients in the carbon source (Singh et al. 2008). This is the first report on *S. obclavatum* MTCC 9604, which produced 3.93 IU ml^-1^ extracellular xylanase after 168 h when wheat bran was used as the substrate in Solid State Fermentation (SSF). Scanning Electron Microscopy revealed the growth of *Simplicillum obclavatum* MTCC 9604 on solid state matrix showing protruding aerial as well as submerged mycelia (Figure 1A). Comparative analysis of xylanase production in SSF and SF revealed that SSF is much more advantageous in terms of xylanase production and total protein secretion. Only 16.6% of soluble protein containing 8.1% of xylanase activity was obtained in case of SF with respect to SSF. Highest xylanase activity was obtained after a 72 h of SF culture (Figure 1B). The highest level of protein secretion was found after 120 hours. After that there was gradual decrease in protein content in SF. This was probably due to protease secretion in SF (Shah and Madamwar 2005). However, liquid fermentation is not a practical method to treat a huge amount of available utilizable biomass. Rather, SSF may have potential industrial applications because of cheap raw material and low cost in downstream processing. The kinetic profile of enzymatic production by *S. obclavatum* MTCC 9604 in SSF using wheat bran as the substrate was obtained after cultivation at 30°C for 7 days. The highest xylanase activity was obtained after a 7 day culture in SSF (Figure 1C).

### SDS-PAGE and zymogram analysis of the expression multiplicity of xylanases

**Figure 2:**
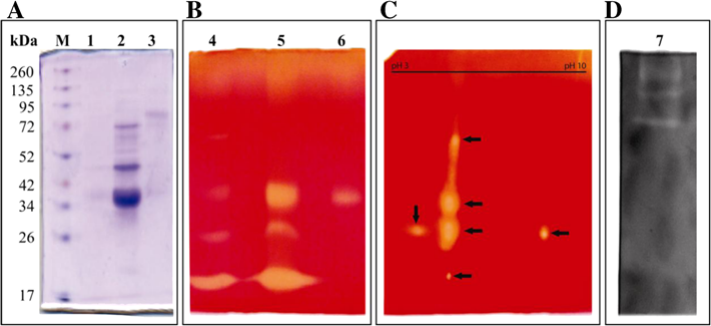
**SDS-PAGE and zymogram analysis of xylanase of*S. obclavatum.*** Panel **(A)** Coomasie stained SDS-PAGE. Lane 1 crude protein, lane 2 partially purified, lane 3 submerged protein extract and lane M molecular weight marker. Panel **(B)** Zymogram of xylanase. Lane 4 crude protein, lane 5 partially purified xylanase from SSF, lane 6 partially purified xylanase from SF. Panel **(C)** 2D zymogram of crude xylanase from SSF. Panel **(D)** lane 7 represents zymogram of endoglucanase from SSF.

After the separation of the enzyme samples by SDS-PAGE containing 2% birchwood xylan, the gel was divided into two parts. One part was subjected to zymogram analysis and other part was stained with Coomassie Brilliant Blue R-250. Figure 2A represents Commassie stained fungal secretome run in 10% SDS PAGE containing xylan (Lane 3). Figure 2B shows that wheat bran induced the production of four distinct isoforms of xylanase (Lane 1) in SSF as evidenced in zymogram analysis, when the crude enzyme extract was run in 10% SDS PAGE. Interestingly, a single xylanase isoform was found to be present in crude enzyme extract obtained from SF (Figure 2B, Lane 6). Liao et al. reported 14 isoforms of xylanase from *Penicillium oxalicum* GZ-2 grown in wheat straw (Liao et al. 2012). Badhan et al. found that in *Myceliophthora sp.* rice straw induced the maximum number (6) of xylanase isoforms, followed by wheat straw (4) (Badhan et al. 2007). Five xylanase isoforms were also found in *Penicillium purpurogenum* grown in oat spelt xylan (Chavez et al. 2002a) and induction of xylanases was found to be dependent on the type and composition of the carbon source used. To investigate the actual number of xylanase isoforms produced by *S. obclavatum* MTCC 9604*,* the crude enzyme extract was run in 2D gel electrophoresis followed by zymogram analysis. Surprisingly, six isoforms of xylanases were visualized among which three isoforms are of similar molecular weight but with different pI in 2D gel (Figure 2C). The production of a multienzyme system of xylanases in which each enzyme has a special function, could be the strategy for microorganisms to achieve effective hydrolysis of xylan. Probably the structural complexity of lignocelluloses has resulted in the need for these multiple forms (Ashok et al. 2011). Interestingly, the environmental conditions have a great impact on the number of isoforms produced by the microorganisms. In the present study same substrate (wheat bran) could induce variation in number of xylanase isoforms produced by *S. obclavatum* MTCC 9604 when grown in SSF (six isoforms) versus SF (single isoform). Various mechanisms have been suggested to account for the multiplicity of function and specificity of xylan degrading enzymes. Electrophoretically distinct xylanases could arise from post translational modification of a gene product such as differential glycosylation or proteolysis. Multiple endoxylanases can also be expressed by distinct alleles of one gene or even by completely separate genes (Ashok et al. 2011). To check the production on endoglucanase in SSF by *S. obclavatum* MTCC 9604, zymogram analysis of crude enzyme extract was performed in presence of carboxymethyl cellulose. Multiple high molecular weight endoglucanases were found to be produced by *S. obclavatum* MTCC 9604 in SSF as evidenced from the zymogram (Figure 2D).

### Partial purification of xylanase obtained from SSF

**Figure 3:**
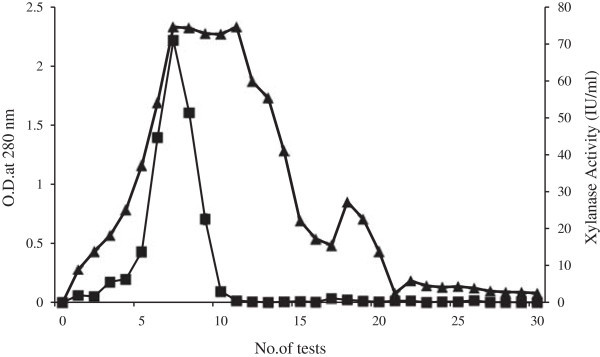
**Partial purification profile of xylanase of*S. obclavatum.*** The bound proteins were eluted from Superose 12 10/300 GL column with 50 mM potassium phosphate buffer pH 7.0 and the fractions were collected at 1 ml volume each. The fractions were assayed for xylanase activity and subjected to determine the absorbance at 280 nm wavelength.

Gel filtration was opted as a purification tool for characterizing of the physicochemical properties of the endoglucanase free xylanase enzyme. Table 1 summarizes the procedure for the purification of the crude enzyme extract obtained from *S. obclavatum* MTCC 9604 grown under SSF conditions. Initial 80% ammonium sulfate cut of the crude extract and successive dialysis resulted in 1.54 fold purification. The final purification step with Superose 12 gel filtration column revealed three major isoforms in the most active 7^th^ fraction. Figure 3 represents the purification profile in Superose 12 column which afforded 2.72% yield with a 24.02 fold purification of the crude enzyme extract collected from the SSF bed. Figure 2A (Lane 2) and Figure 2B (Lane 5) represent the Commassie stained and zymogram profile of the purified fraction respectively. An excess amount of protein was loaded in gel stained with Commassie blue to observe the purity of the fraction (Figure 2A, Lane 2). One high molecular weight xylanase isoform was found to be reduced during gel filtration as evidenced from the zymogram analysis.

**Table 1:** **Partial Purification profile of xylanase of*S. obclavatum*from SSF**

Purification step	Total protein (mg)	Xylanase total activity (μ mol/min/ml)	Specific activity (μ mol/min/mg)	Purification fold	% Yield
Crude	20	236.4	591	1	100
Ammonium sulfate precipitation	8.8	79.92	908.2	1.54	44
Superose 12 FPLC	0.543	71.0	14200	24.02	2.72

### Effect of pH on xylanase activity and stability

**Figure 4:**
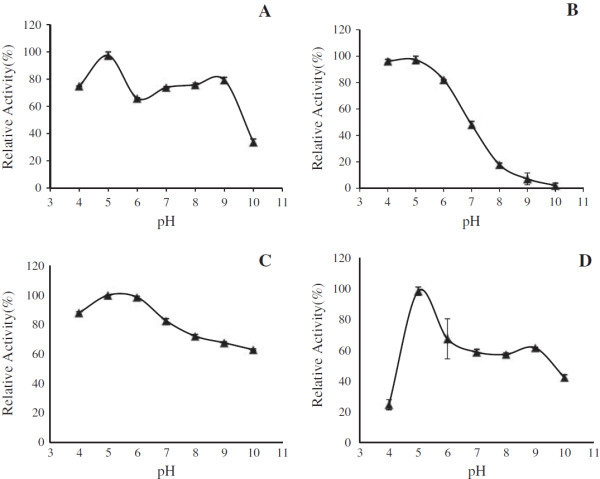
**pH optima and pH stability curve of xylanase of*S. obclavatum*from SSF and SF.** Panel **(A)** pH optima curve of xylanase from SSF. Panel **(B)** pH optima curve of xylanase from SF. Panel **(C)** pH stability curve of xylanase from SSF. Panel **(D)** pH stability curve of xylanase from SF. Relative enzyme activities (% of maximum) were plotted against pH. All the results were expressed in mean ± SD from n = 3.

Partially purified xylanase exhibited the highest xylanase activity at pH 5 and retained 80% activity at the pH 9 (Figure 4A). This data corroborated well with 2D zymogram where a distinct isoform was present at around pH 9. Four isoforms were found to be present at pH 5. One isoform was present at around ~ pH 4 in 2D zymogram. On the other hand the Xylanase produced from SF condition showed the optimum activity (1.55 IU/min) at pH 5 and then the activity gradually decreased down at alkaline condition (Figure 4B). The partially purified xylanases from S*. obclavatum* MTCC 9604 grown in SSF were highly stable within a broad pH range, ranging from pH 4.0 to 9.0 (Figure 4C). Thus, the pH stability of xylanase obtained from *S. obclavatum* MTCC 9604 showed to be a promising fungus for potential biotechnological applications. The xylanase produced in SF also showed a wide range of pH stability starting from pH 5.0 to 9.0 (Figure 4D).

### Effect of temperature on xylanase activity and stability

**Figure 5:**
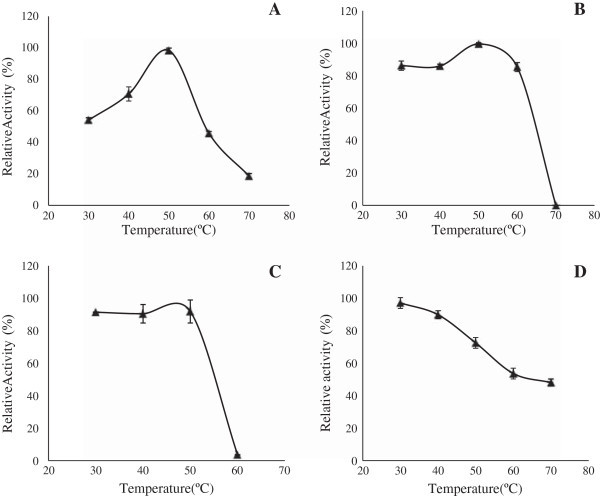
**Temperature optima and thermal stability curve of xylanase of*S. obclavatum*from SSF and SF.** Panel **(A)** Temperature optima of xylanase from SSF. Panel **(B)** Temperature optima of xylanase from SF. Panel **(C)** Thermal stability of the xylanase from SSF. Panel **(D)** Thermal stability of the xylanase from SF. Relative enzyme activities (% of maximum) were plotted against temperature. All the results were expressed in mean ± SD from n = 3.

The optimum temperature of the purified xylanase from both SSF and SF was found to be 50°C at pH 7.0 (Figure 5A). Partially purified xylanases produced from SSF were more thermo stable with compare to the xylanases produced from SF condition. The partially purified xylanases from SSF exhibited stability over the temperature range of 30-60°C and retained about 80% residual activity on that range (Figure 5B). Whereas the xylanase produced in SF gradually lost its activity and retained only 50% residual activity after 1 h incubation at 60°C (Figure 5B).

### Effect of metal ions and other compounds on xylanase from SSF culture condition

Different metal ions showed varied effect (Table 2) according to the nature of the chemicals. Addition of the 3 mM and 6 mM of Mg^++^, Ni^++^, Cu^++^ and Hg^++^ caused strong inhibition of enzyme activity, whereas 3 mM concentration of Zn^++^ activate the Xylanase activity. The enzymes can be modulated by the interaction of cations with the amino acid residues involved in their active sites. This type of interaction can either positively or negatively modulate the enzyme activity (Dutta et al. 2006; Dutta et al. 2008). The enzyme activity get hampered in presence of EDTA, indicating that the xylanase contains any types of metal ion in its active site on the other hand NaCl have mild enhancing effect on the xylanase. Nonionic detergent like triton X-100 has mild inhibitory effect on enzyme activity. The strong elevation of the enzyme activity in the presence of thiol group protecting reagents like β-marcapto ethanol and cysteine can be explained by preventing the oxidation of the sulfhydryl groups in presence of the same. The explanation for the stimulatory effects by tryptophan may be because of the alteration of enzyme conformation (Dutta et al. 2006; Dutta et al. 2008). Further studies are required to elucidate the structure-function of these xylanases.

**Table 2:** **Effect of metal ions and other compounds on xylanase of*S. obclavatum*from SSF**

Agents	Relative activity of Xylanase at 3 mM	Relative activity of Xylanase at 6 mM
Control	100	100
DTT	371	472
Mg^++^	Negligible	32.58
Hg^++^	Negligible	Negligible
SDS	110	152
TRITON X100	54	63
TRYPTOPHAN	354	353
L-CYSTEINE	250	202
Zn^++^	148	151
EDTA	79	64
BME	437	442
NaCl	160	120
Ni	Negligible	34
Cu^++^	Negligible	Negligible

The activity is expressed as a percentage of the activity level in the absence of chemicals (control).

## Conclusions

*S. obclavatum* MTCC 9604 produces multiple xylanolytic enzymes in solid state fermentation using wheat bran as inducer. Apart from the production of industrial enzyme in SSF, the studies on this particular microorganism may also have mycological significance. This study clearly showed that environmental factors dictate extracellular protein secretion and isoform variation. Nonetheless, xylan degrading enzymes produced in solid state culture were remarkably stable towards a wide range of pH and temperature which make them potentially suitable for applications in different industries.

## Material and methods

### Reagents

All the reagents were analytical grade and purchased from Sigma Chemical Co (St Louis, MO, USA) unless specified.

### Microorganism

White, long, obclavate xylanase producing fungi was isolated from soil in our laboratory. The strain was sent to Institute of Microbial Technology (IMTECH) for identification. IMTECH identified the culture as *Simplicillium obclavatum* MTCC 9604 which was used for the production of xylanase. The fungal colonies were reached up to 30–35 mm in diameter in potato dextrose agar (PDA) plates after seven days of incubation at 30°C. The microorganism was maintained over PDA and it was cultivated for the xylanase production in both submerged and solid state fermentation described below.

### Production of xylanase in submerged fermentation

Spores were collected from PDA slants in Mandels mineral salt solution (Mandels et al. 1974). The composition of Mandels mineral salt solution was as follows: H_2_O 495 ml, concentrated HCl 5 ml, FeSO_4_, 7H_2_O, 4.6, MnSO_4_, H_2_O, 0.89, ZnSO_4_, H_2_O, 1.78, CoCl_2,_ 1.25. For submerged fermentation, 10^4^ -10^6^ spores of *S. obclavatum* was grown in 50 ml liquid medium containing wheat bran as sole source of carbon. Medium composition (g L^-1^) was as follows: 1.4 (NH_4_)_2_ SO_4_, 0.3 Urea, 2 KH_2_PO_4_, 0.3 MgSO_4_ 7H_2_O, 0.3 CaCl_2_, 0.4% Trace Metal (g ml^-1^). The submerged culture was incubated at 30°C for 3 days at 150 rpm shaking condition. After 3 days, culture filtrate was collected by filtration. Extracellular xylanase activity was measured in the culture filtrate of the submerged fermentation.

### Partial purification of xylanase from submerged fermentation

All purification steps were performed at 4°C unless otherwise stated. The culture filtrate was subjected to 0-80% (NH_4_)_2_SO_4_ saturation. At 80% salt saturation, no enzyme activity remained in the supernatant as all had been recovered in the precipitate. The precipitated protein collected by centrifugation at 10, 000 rpm was dissolved in minimum volume of 50 mM potassium phosphate buffer pH 7.2 and then it was dialyzed against the same buffer. The dialysate was used for xylanase assay.

### Production of xylanase in solid state fermentation

Spores were collected from PDA slants in Mandels mineral salt solution and spread over the solid bed (10^4^ -10^6^ spores per 5.0 g of wheat bran) uniformly maintaining the moisture content of the solid bed of about 50% with Mandel’s salt medium. Mandels salt medium was homogeneously mixed with active inoculum and incubated at 30°C for 7 days. Fermented substrates were extracted at different time interval to optimize the culture incubation time in the solid bed. After proper incubation, the enzyme was extracted from wheat bran by agitating it in 20 ml of 50 mM potassium phosphate buffer, pH-7.2 for 1 h in a shaker with 150 rpm. Fermented substrates were separated from the extraction buffer by filtration through cheese- cloth. Supernatant was collected from the agitated mixture after a centrifugation at 12000 rpm for 30 min at 4°C. After that the collected supernatant was subjected to partial purification.

### Partial purification of xylanase from SSF

All purification steps were performed at 4°C unless otherwise stated. Finely powdered (NH_4_)_2_SO_4_ was added to the enzyme extract to 80% saturation. After 2 h on ice, the precipitate formed was collected by centrifugation at 10, 000 rpm, dissolved in 50 mM potassium phosphate buffer pH 7.2 and dialyzed against the same buffer. At 80% salt saturation, almost no enzyme activity remained in the supernatant as all had been recovered in the precipitate. After dialysis, the non-dissolved protein was removed by centrifugation at 10,000 rpm for 10 min at 4°C and the clear supernatant was applied to a FPLC Superose 12 10/300 GL (Amersham Pharmacia, Uppsala, Sweden) pre equilibrated with 50 mM potassium phosphate buffer pH 7.2. The enzyme was eluted in the same buffer with a constant flow rate. The elution of the protein was monitored at 280 nm. Protein concentration was determined according to the method of Bradford (Bradford 1976). The active fractions were collected and kept at −80°C and used further for xylanase assay and zymogram analysis.

### Xylanase assay

All xylanase assays were performed with 50 mM potassium phosphate buffer pH −7.2 unless otherwise specified. Xylanase activity was determined as per the International Union of Pure and Applied Chemistry recommendations, with 10% xylan as the substrate. The xylanase assay was carried out by incubating appropriately diluted enzyme in 50 mM potassium phosphate buffer pH −7.2 with 10% birchwood xylan (w/v) at 50°C for 30 min. The released reducing sugar was determined by dinitrosalicylic acid (DNS) method using D-xylose as standard (Miller 1959). Soluble xylan was prepared by suspending 4 g of birchwood xylan in 100 ml of 50 mM K_2_HPO_4_/KOH buffer, pH-7.0, and stirring the suspension for 4 h followed by centrifugation. The soluble fraction corresponds to 50% (w/v) of the total xylan (Yang et al. 1995). One unit of xylanase activity was defined as the amount of enzyme that produced 1 μmol of xylose equivalent per min from xylan under assay conditions.

### SDS polyacrylamide gel electrophoresis and zymogram analysis

SDS polyacrylamide gel electrophoresis (SDS-PAGE) analyses were performed using 10% (w/v) polyacrylamide gel with a 4% stacking gel and the Mini-Protean II system (BioRad) according to the method described by Laemmli, with some modifications (Laemmli 1970). Protein bands were visualized by staining by Coomassie Brilliant Blue. The zymogram analysis of xylanase from SSF culture was performed according to Tseng et al. (Tseng et al. 2002). After the separation of the enzyme samples by SDS-PAGE containing 2% birchwood xylan, the gel was divided into two parts. One part was processed for protein staining with Coomassie Brilliant Blue and the other part was used for zymogram analysis. For zymogram analysis, the gel was washed first with 50 mM potassium phosphate buffer pH −7.2 containing 25% isopropanol and the gel was kept in the same solution for 1 h at room temperature. The gel was again washed with 50 mM potassium phosphate buffer pH −7.2 without isopropanol and it was kept in the same buffer for 30 min at room temperature. After that the gel was incubated at 37°C for 10 min. Finally the gel was stained with 0.1% Congo red solution followed by destaining with 1 M NaCl solution until pale-red hydrolysis zones appeared against a red background. The molecular weights of the isoforms were determined by correlating it with identical SDS –PAGE profile of protein molecular weight standard.

### 2D gel electrophoresis followed by zymogram analysis of xylanase

2D gel electrophoresis of xylanase was performed with the Ettan IPGphor II system (GE healthcare limited, Buckinghamshire UK). For the first dimension 7 cm NL, pH 3–10, immobilized strips were used where 250 μg of protein was loaded together with the rehydration buffer containing 8 M urea, 2% (v/v) triton x 100, 0.28% (w/v) DTT, 1% (v/v) IPG buffer containing ampholytes and 0.002% (w/v) bromophenol blue (all final concentrations). For the preparation of sample, 250 μg proteins were directly solubilized in the above rehydration buffer and subjected to two consecutive freeze thaws for complete lysis in presence of protease inhibitor cocktail. IPG strips were rehydrated with samples at 50 μA/strip for 12 h at 20°C and then isoelectric focusing was performed at 25°C by a linear increase to 500 V for 2 h followed by a gradient increase to 4500 V over 1 h and then held at 4500 V until a total of 14 KVH was reached. For the second dimension the IPG strips were first equilibrated for 15 min in SDS equilibrium buffer containing 50 mM Tris–HCl (pH 8.8), 6 M urea, 30% (v/v) glycerol, 2% (w/v) SDS, 1% (w/v) DTT and 0.002% (w/v) bromophenol blue and then in 50 mM Tris–HCl (pH 8.8), 6 M urea (w/v), 30% glycerol (v/v), 2% SDS (w/v), 2% iodoacetamide (w/v) and 0.002% (w/v) bromophenol blue for another 15 min. The strips were then embedded in 0.5% (W/V) agarose on top of a 10% acrylamide gel with 2% xylan as a substrate containing 3% stacking gel. Zymogram analysis of xylanase was performed according to Tseng et al.

### Effect of pH and temperature on xylanase

To determine the optimum pH of xylanase, 10% xylan was taken in buffers of different pH for the experimental purpose. The partially purified enzyme either from SSF or SF was then added to the buffer of different pH containing substrate and the reaction mixture was incubated for 30 min at 50°C. Liberated reducing sugar was then estimated by 3, 5-Dinitrosalicylic acid (DNS) reagent.

For pH stability experiment, the enzyme was exposed for 1 h at 25°C to the corresponding buffer. The residual activity of the enzyme was measured in 50 mM potassium phosphate buffer, pH 7.2. The following buffer solutions were employed to determine the pH optima and pH stability curve: 50 mM sodium acetate buffer (pH 4–5), 50 mM potassium phosphate buffer (pH 6–8), and 50 mM Glycine-NaOH buffer (pH 9–10).

To determine the optimum temperature of xylanase, partially purified enzyme either from SSF or SF was incubated with 10% of the substrate at different temperature for 30 min in 50 mM potassium phosphate buffer, pH 7.2. The liberated sugar was then estimated by 3, 5-Dinitrosalicylic acid (DNS) reagent.

Thermostability of xylanase was determined by incubating the partially purified enzyme either from SSF or SF in 50 mM potassium phosphate buffer, pH 7.2 at different temperature for 1 h. After incubation it was subjected to centrifugation to precipitate the denatured protein which may generate due to thermal denaturation. The enzyme was diluted appropriately before the assay. The residual activity of the enzyme was measured in 50 mM potassium phosphate buffer, pH 7.2 by Dinitrosalicylic acid (DNS) reagent.

### Effect of metal ions and other compounds on xylanase from SSF condition

To investigate the effect of metal ions, EDTA, SDS, triton-X100, DTT, β-mercaptoethanol (BME), NaCl, L-tryptophan and L-cysteine on the enzyme activity, 3 mM and 6 mM concentration of those compounds were used in the assay mixture and the % residual enzyme activities were measured by DNS reagent.
